# Functional Role of Kallikrein 6 in Regulating Immune Cell Survival

**DOI:** 10.1371/journal.pone.0018376

**Published:** 2011-03-28

**Authors:** Isobel A. Scarisbrick, Benjamin Epstein, Beth A. Cloud, Hyesook Yoon, Jianmin Wu, Danielle N. Renner, Sachiko I. Blaber, Michael Blaber, Alexander G. Vandell, Alexandra L. Bryson

**Affiliations:** 1 Neurobiology of Disease Program, Mayo Medical and Graduate School, Rochester, Minnesota, United States of America; 2 Department of Physical Medicine and Rehabilitation, Mayo Medical and Graduate School, Rochester, Minnesota, United States of America; 3 Department of Neurology, Mayo Medical and Graduate School, Rochester, Minnesota, United States of America; 4 Department of Biomedical Sciences, Florida State University College of Medicine, Tallahassee, Florida, United States of America; Universidade de Sao Paulo, Brazil

## Abstract

**Background:**

Kallikrein 6 (KLK6) is a newly identified member of the kallikrein family of secreted serine proteases that prior studies indicate is elevated at sites of central nervous system (CNS) inflammation and which shows regulated expression with T cell activation. Notably, KLK6 is also elevated in the serum of multiple sclerosis (MS) patients however its potential roles in immune function are unknown. Herein we specifically examine whether KLK6 alters immune cell survival and the possible mechanism by which this may occur.

**Methodology/Principal Findings:**

Using murine whole splenocyte preparations and the human Jurkat T cell line we demonstrate that KLK6 robustly supports cell survival across a range of cell death paradigms. Recombinant KLK6 was shown to significantly reduce cell death under resting conditions and in response to camptothecin, dexamethasone, staurosporine and Fas-ligand. Moreover, KLK6-over expression in Jurkat T cells was shown to generate parallel pro-survival effects. In mixed splenocyte populations the vigorous immune cell survival promoting effects of KLK6 were shown to include both T and B lymphocytes, to occur with as little as 5 minutes of treatment, and to involve up regulation of the pro-survival protein B-cell lymphoma-extra large (Bcl-XL), and inhibition of the pro-apoptotic protein Bcl-2-interacting mediator of cell death (Bim). The ability of KLK6 to promote survival of splenic T cells was also shown to be absent in cell preparations derived from PAR1 deficient mice.

**Conclusion/Significance:**

KLK6 promotes lymphocyte survival by a mechanism that depends in part on activation of PAR1. These findings point to a novel molecular mechanism regulating lymphocyte survival that is likely to have relevance to a range of immunological responses that depend on apoptosis for immune clearance and maintenance of homeostasis.

## Introduction

Kallikrein 6 (KLK6) is a secreted serine protease and member of the largest contiguous cluster of serine proteases in the human genome. In prior studies we have demonstrated elevated levels of KLK6 occur in active MS lesions and in the serum of patients experiencing a secondary progressive disease course. KLK6 levels have also been shown to be present in infiltrating immune cells at sites of CNS inflammation in animal models of MS and to be up regulated in T cells with activation [1], [2], [3], [4]. Additionally, blocking KLK6 activity diminishes clinical and histological disease as well as Th1 inflammatory responses in experimental autoimmune encephalomyelitis (EAE) [5], yet the role and mechanism of action of KLK6 in immune function has not been previously examined.

While best characterized for its ability to degrade extracellular matrix factors including laminin, fibronectin and heat denatured collagen [6], [7], in addition to myelin proteins [1], KLK6 has also been recently demonstrated to activate a subset of protease activated receptors (PAR) [8], [9], [10], [11]. PARs are G-protein coupled transmembrane receptors that are uniquely activated by proteolytic cleavage and well known to play key roles in driving inflammatory responses. Of the four known PARs, KLK6 has been shown to activate PAR1 in neurons and both PAR1 and PAR2 in astrocytes triggering intracellular Ca^2+^ flux and differentially affecting both MAPK and AKT signaling pathways [11]. KLK6 was also shown to activate PAR2 in HEK cells to trigger Ca^2+^ signaling and on aortic endothelial cells to mediate relaxation [9]. Whether KLK6 induces signaling in lymphocytes and the dependence of this on PAR has not been previously addressed.

The physiological actions of PARs have perhaps been most extensively studied in terms of their regulation of immune responses. PAR-specific activities have been described in both innate and adaptive immunity, including roles in mast cells, eosinophils, basophils, monocytes, dendritic cells and T cells (see [12] for review). For example, mast cell tryptase, known to activate PAR2, promotes synthesis and release of pro-inflammatory cytokines from peripheral blood mononuclear cells [13]. PAR1 and -2 activation in monocytes and dendritic cells results in increased GM-CSF and IL-6, but reduced IL-4 [14], [15], [16]. In T and B cells, PAR1 activation promotes proliferation and augments T cell CD3 and IL-2 responses [17]. Studies using PAR deficient mice indicate a duality of PAR function depending on the context with both pro- and anti-inflammatory roles being reported. For example, in rodents PAR1 has been shown to play an anti-inflammatory role in cases of *H. pylori* induced gastritis [18], but exacerbates inflammation in a murine model of colitis [19], crescentic glomerulonephritis [20], and BSA induced arthritis [21]. PAR2 has been demonstrated to play an anti-inflammatory role in airways [22] and intestine [19], but a role in disease exacerbation in adjuvant induced arthritis [23], allergic dermatitis [24] and in EAE [25].

Given the abundant expression of KLK6 by T cells and monocytes at sites of CNS inflammation, in the serum of patients with secondary progressive MS, and its regulation by immune cell activation, determination the likely role(s) of this unique serine protease in immune function is long overdue. Herein we examine the effect of KLK6 on lymphocyte survival and the possible role of PAR1. Results indicate KLK6 promotes survival of murine splenocytes by a mechanism that depends in part on activation of PAR1 and which involves at least in part the up regulation of pro-survival and down regulation pro-apoptotic Bcl-2 family member signaling proteins.

## Materials and Methods

### Ethics Statement

All animal experiments were carried out with strict adherence to NIH Guidelines for animal care and safety and were approved by the Mayo Clinic Institutional Animal Care and Use Committee, Animal Welfare Assurance number A3291-01, and Protocol number A28508.

### Model Systems

The effect of KLK6 on immune cell survival was examined using whole splenocyte populations derived from C57BL6/J or PAR1 deficient mice or the Jurkat T-leukemic cell line (clone E6-1, TIB-152 American Type Culture Collection). Eight to 12 week old C57 mice were obtained from Jackson Laboratories. Mice deficient in PAR1^−/−^ (B6.129S4-*F2r^tm1Ajc^*/J) were also obtained from Jackson and backcrossed to C57BL6/J for 10 generations, such that C57 mice served as wild type controls in each experiment. All animal experiments were carried out with strict adherence to NIH Guidelines for animal care and safety and were approved by the Mayo Clinic Institutional Animal Care and Use Committee.

### Cell Culture

Spleens were homogenized in RPMI-1640, red blood cells lysed with ammonium chloride buffer, and splenocytes cultured in tissue culture treated 96 well plates at a density of 7.5×10^5^ cells/ml. All experiments were performed in serum free X-Vivo media (Lonza, Mapleton, IL) containing 2 mM Glutamax, 1 mM sodium pyruvate, 50 U/mL penicillin-streptomycin, 10 mM HEPES, 50 µM 2-β-mercaptoethanol and 10 µM non-essential amino acids (Invitrogen). Jurkat T cells were maintained in log-phase growth in the same defined media. All cells were maintained at 37°C in 95% air and 5% CO_2_. All culture conditions were examined in triplicate within a given experiment and all experiments repeated at least twice.

### Reagents

Recombinant KLK1 and KLK6 were expressed using a baculovirus/insect system, purified and activated as previously described in detail [6], [7], [26]. We have developed expression systems for both *Homo sapiens* and *Rattus norvegicus* KLK6 [6], [7]. Phylogenetic analysis identifies *Homo sapiens* KLK6 and Rattus norvegicus Klk6 as unambiguous orthologs [6]. In a pair-wise comparison of the KLK6 proteins of these species there are no insertions or deletions of amino acids. The mature proteins are 223 amino acids in length in each case. The functional activity of *Rattus norvegicus* and *Homo sapiens* KLK6 mature proteins can been directly compared for digestions of myelin basic protein, laminin and fibronectin and these show essentially identical specificities; and the conservation of the regulatory autolytic inactivation property for each protein has also been demonstrated [6], [7]. The characteristic preference for arginine versus lysine in the substrate P1 position has also been directly compared between *Rattus norvegicus* and *Homo sapiens* KLK6 mature proteins using identical tripeptide substrates and has been demonstrated to be highly-conserved in these species orthologs. *Mus musculus* KLK6 contains two additional amino acids at the C-terminus but since this is anti-podal to the active site it would have no known effect upon enzyme activity or specificity and therefore is predicted to be identical in function to the human and rat forms. Thus, the available evidence supports a highly-conserved sequence, functional activity, and substrate specificity between mouse, rat and human KLK6 orthologs. The primary observation in this report, that KLK6 improves survival of murine splenocytes and the human Jurkat T cell line, has been observed in experiments which utilized either the *Rattus norvegicus* or *Homo sapiens* forms of KLK6, and all experiments shown were carried out using *Rattus norvegicus* KLK6. All experiments involving KLK1 utilized highly purified recombinant *Homo sapiens* KLK1 [26]. Use of *Homo sapiens* KLK1 to study physiological effects in a murine system has been previously validated [27]. The functional activity of recombinant KLKs were examined at concentrations ranging from 1 to 10 µg/ml (40 to 400 nM) which correspond to concentrations that our prior studies have shown induce intracellular signaling [11]. The concentrations of KLK6 examined also encompass the physiologic level seen in normal cerebrospinal fluid (0.5–2 µg/ml) [28], [29], [30] and up to 5-fold excess, which we anticipate partially models the elevated levels seen at sites of CNS inflammation in MS and its animal models [1], [2], [3], [4].

### KLK6-Over Expression

Results demonstrated using recombinant KLK6 were confirmed in Jurkat T cells using a KLK6 over expression system. Jurkat T cells were transduced with a vector in which the *Homo sapiens* KLK6 gene (NM_002774) is under the control of a cytomegalovirus (CMV) promoter (GeneCopoeia, Inc., Rockville, MD), or the corresponding empty vector, by electroporation using a Genetronics BTX T820 square-wave electroporator (San Diego, CA). Stably transduced Jurkat cells were selected using 0.4 mg/ml G418 (Invitrogen, Carlsbad, CA). All experiments described were carried out in serum free X-Vivo media without the addition of G418. Levels of KLK6 over expression in stable cell lines were quantified by real time PCR as previously described in detail [3]. Briefly, total RNA was extracted from G418-selected Jurkat cells transduced with KLK6-CMV, or vector alone, using RNA STAT-60 (Tel-Test, Inc. Friendswood, TX) and 0.5 µg of total RNA subject to RT-PCR using the Light Cycler-RNA Amplification Kit SYBR Green I (Roche Diagnostics, Mannheim, Germany) and an i-Cycler IQ Real-Time PCR instrument (BioRad, Hercules, CA). KLK6 expression levels were normalized to those for glyceraldehyde phosphate 3-dehydrogenase (GAPDH). Forward and reverse primers for *Homo sapiens* KLK6 were Forward, 5′-TGCCAGGGTGATTCTGGG-3′ and Reverse 5′-TGCAGACGTTGGTGTAGACT-3′; and for GAPDH were Forward 5′-ACCACCATGGAGAAGGC-3′ and Reverse 5′-GGCATGGACTGTGGTCATGA-3′. Expression levels in each case were quantified relative to standard curves resulting from amplification of KLK6 or GAPDH nucleic acid templates.

### Cell Death Paradigms

To determine the scope of action of KLK6 in altering immune cell survival we examined its effects in a range of cell death paradigms, including those mediated by transmembrane death receptors, such as the Fas receptor (extrinsic pathway), and those which result from cellular stress and activation of intracellular mitochondrial signaling cascades (intrinsic pathways)[31]. Signaling through the Fas receptor is known to directly activate apoptosis by recruitment and activation of caspase enzymes [32] and was induced in the present study using Fas ligand/TNFSF6 (2 µg/ml) cross linked with anti-polyHistidine (10 µg/ml). Several activators of intrinsic apoptotic mechanisms were also examined, including spontaneous (resting) cell death, known to occur after harvest and plating of splenocytes *in vitro* [33] and staurosporine-induced cell death (0.1 to 1 µM), which occurs by broad spectrum protein kinase inhibition [34], [35]. Other activators of intrinsic apoptotic mechanisms were also examined such as the glucocorticoid dexamethasone (0.1 µM), which is widely used as an anti-inflammatory and T cell apoptotic agent [36], [37] and the topoisomerase inhibitor camptothecin (1.0 µM), which was originally developed as an anti-cancer therapeutic. Concanavalin A (ConA, 5 µg/ml) is a lectin and well known lymphocyte mitogen that promotes activation induced cell death [38]. Since camptothecin-induced lymphocyte apoptosis is enhanced in the presence of mitogens [39], we examined the effects of KLK6 on cell death induced by either camptothecin or ConA alone, or in combination. All agents utilized to induce cell death were obtained from Sigma and were either applied to cultures at the time of cell plating simultaneously with KLK6, or after cultures had first been pre-incubated with KLK6 for 2 hr.

### Flow Cytometry

Following experimental incubation periods which ranged from 4 to 72 hr, cells were harvested and stained with combinations of antibodies recognizing to CD45, CD3, B220 or the early apoptotic marker Annexin-V, conjugated to FITC, PE, or APC (e-Bioscience, San Diego, CA). In each case, either propidium iodide (PI) or 7-Aminoactinomycin D (7AAD) were used to label dead cells (Sigma). Examination of the composition of the splenocyte cultures examined revealed them to be predominantly lymphocytes with 24% being CD3^+^ T lymphocytes and 60% being B220^+^ B lymphocytes, while less than 2.5% were CD11b^+^ monocytes. Cells were analyzed by flow cytometry using a FACSCalibur flow cytometer (BD Biosciences, Mountain View, CA). Live immune cells were defined as those which were positive for the common leukocyte antigen CD45, but negative for PI. Early and late apoptotic/dead cells were distinguished using a combination of AnnexinV-Phycoerythrin (PE) and 7AAD or AnnexinV-Fluorescein isothiocyanate (FITC) and PI (BD Biosciences). FlowJo software (Ashland, OR) was used to quantify different cell populations in flow cytometry experiments. Potential effects on cell proliferation were examined by labeling cells with carboxyfluorescein succinimidyl ester [40], [41] (CFSE, Invitrogen, Carlsbad, CA) prior to plating followed by analysis using the FlowJo proliferation platform at the completion of each experiment.

To compare multiple cell culture conditions within a given experiment, the mean and standard error (SEM) across experimental groups were analyzed by One-Way Analysis of Variance (ANOVA) followed by Student-Newman-Keuls (SNK) post hoc test except when data were not normally distributed when ANOVA on Ranks and Mann-Whitney-U analyses were performed. Statistical differences between two groups were compared using the Student's t-test or the Rank Sum test when data were non-linear. P<0.05 was considered statistically significant. All samples were analyzed in triplicate and experiments repeated at least 3 separate times, using independent cell culture preparations, with parallel results.

### Western Blot Analysis

Protein lysates from whole splenocyte cell cultures were separated on SDS-polyacrylaminde gels prior to transfer to nitrocellulose membranes. Antibodies to detect Bcl-XL and Bim were obtained from Cell Signaling Technology (Danvers, MA). The Poly ADP-ribose polymerase (PARP) antibody was a kind gift from Dr. S. Kauffman [42]. Equal loading was verified by re-probing blots for β-Actin (Novus, Littleton, CO). In all cases proteins of interest were detected on film using chemiluminescence Supersignal (Pierce, Rockford, IL). For quantification, films were scanned and images quantified using Image Lab 2.0 software (BioRad). Relative changes in BIM and BCL-XL protein levels in resting or treated cells were determined by normalizing optical density measurements for either protein with those for Actin detected on the same membrane. In the case of PARP, in which level of cleavage was the end point of interest, both the upper band corresponding to 116 kDa intact form, and the signature 89-kDa caspase-generated fragment [43] were each normalized to Actin. The percent PARP cleavage was then determined by calculating the percent that the 89-kDa fragment was of the total PARP (116+89 kDa fragments) detected. All Western blots were repeated at least twice using separate cell culture preparations with similar results.

## Results

### KLK6 differentially affects immune cell survival and proliferation

**Figure 1:**
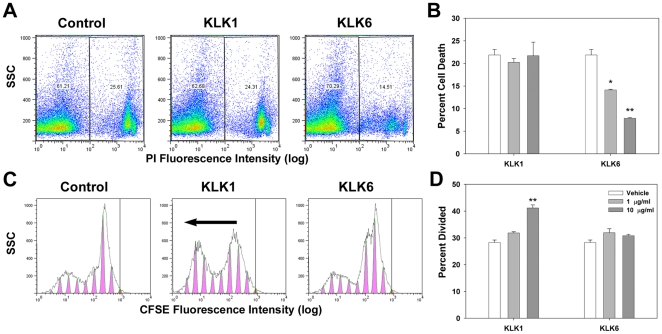
Differential effects of KLK1 and KLK6 on survival and proliferation of murine splenocytes. To differentiate between possible effects of KLK6 on cell survival versus proliferation, murine splenocytes were labeled with CFSE and cultured in defined media in the presence of 1 or 10 µg/ml of KLK1, KLK6, or vehicle alone (Control), for periods of 24 or 72 hr. At harvest dead cells were labeled using PI and samples were examined by flow cytometry. A, Stimulation of cultures with either 1 or 10 µg/ml of KLK6, but not KLK1, significantly reduced the number of dead (PI^+^) cells observed at either the 24 or 72 hr time points (72 hr shown, B). The intensity of CFSE labeling in PI^-^ cells was determined using the proliferation platform of the Flow Jo Program and labeling peaks observed after 24 hr are shown in (C). KLK1 (10 µg/ml), but not KLK6 promoted a significant increase in the percent of cells divided at the 24 hr time point (D); Data are expressed as mean ± SEM, One Way ANOVA with SNK post hoc test; P<0.001**, P<0.02*. (SSC, side scatter). Parallel observations have been made in a least three separate cell culture experiments.

**Figure 2:**
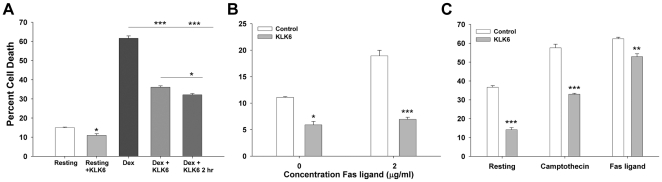
KLK6 decreases death of murine splenocytes and Jurkat T cells across multiple paradigms. KLK6 (10 µg/ml) significantly reduced the number of PI^+^ dead splenocytes observed by flow cytometry after a 24 hr period in culture under resting conditions, in response to (0.1 µM) dexamethasone (A), or after 24 hr exposure to Fas ligand (FasL, 2 µg/ml, B). In parallel experiments (C), Jurkat T cells showed significantly reduced levels of cell death under resting conditions and in response to camptothecin (1.0 µM), or Fas ligand (C, 2 µg/ml) when co-treated with KLK6 (10 µg/ml). Fas ligand at 2 µg/ml caused greater cell death in Jurkat cells (C) relative to splenocytes (B) under the conditions of this study. KLK6 was applied at the time of plating in conjunction with cell death inducing agents in each case. A small but significant improvement in the ability of KLK6 to rescue splenocytes from death was seen with a 2 hr pre-incubation prior to the addition of dexamethasone (A); Data are expressed as mean ± SEM of triplicate cultures examined in parallel, One Way ANOVA with SNK post hoc test for multiple comparisons in (A), two-way comparisons A to C, were made using Students t-test; P<0.001***, P≤0.005**, P≤0.02*. All results shown are representative of at least 3 independent cell culture experiments.

**Figure 3:**
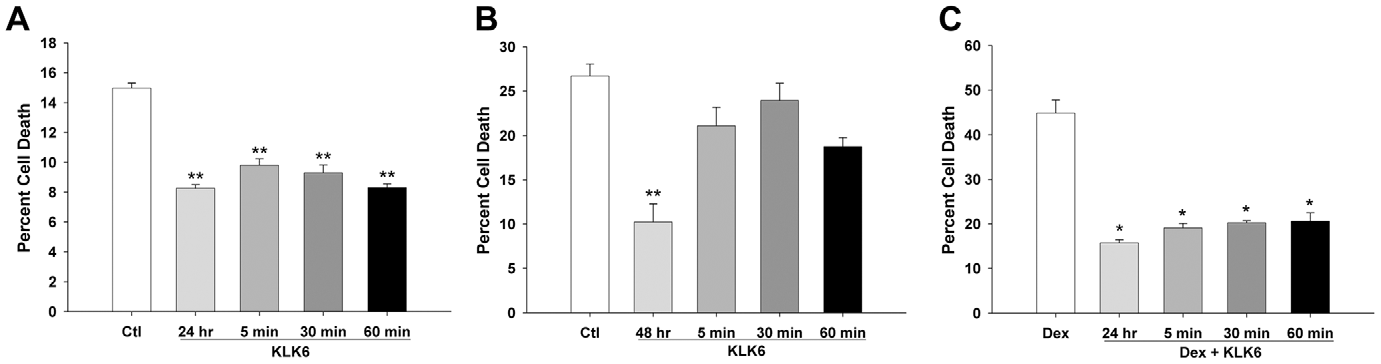
As little as a 5 minute pulse of KLK6 is sufficient to promote splenocyte survival. To gauge the minimal time of exposure to KLK6 necessary to observe its pro-survival effects, splenocytes were pulsed with KLK6 (10 µg/ml) for periods of 5, 30 or 60 min, or in the presence of KLK6 for the full 24 or 48 hr period of culture examined, prior to labeling with PI for analysis of dead cells by flow cytometry (A and B). A 5 min pulse with KLK6 promoted significant survival of explanted resting splenocytes over a 24 hr culture period that was similar in magnitude to that seen with longer pulses, i.e. 30 or 60 min and 24 hr (A). A 5 min pulse with KLK6 was also sufficient to promote survival when cells were exposed to dexamethasone (0.1 µM) for 24 hr (C). After longer periods of culture however (B, 48 hr), only more the more prolonged period of KLK6 stimulation (48 hr) exerted significant pro-survival effects (B). Data shown are the mean ± SEM of triplicate cell culture samples run in parallel and examined by One Way ANOVA with the SNK post hoc test for multiple comparisons; P<0.001**, P≤0.005*. Results shown are representative of at least 3 separate experiments using independent cell culture preparations.

**Figure 4:**
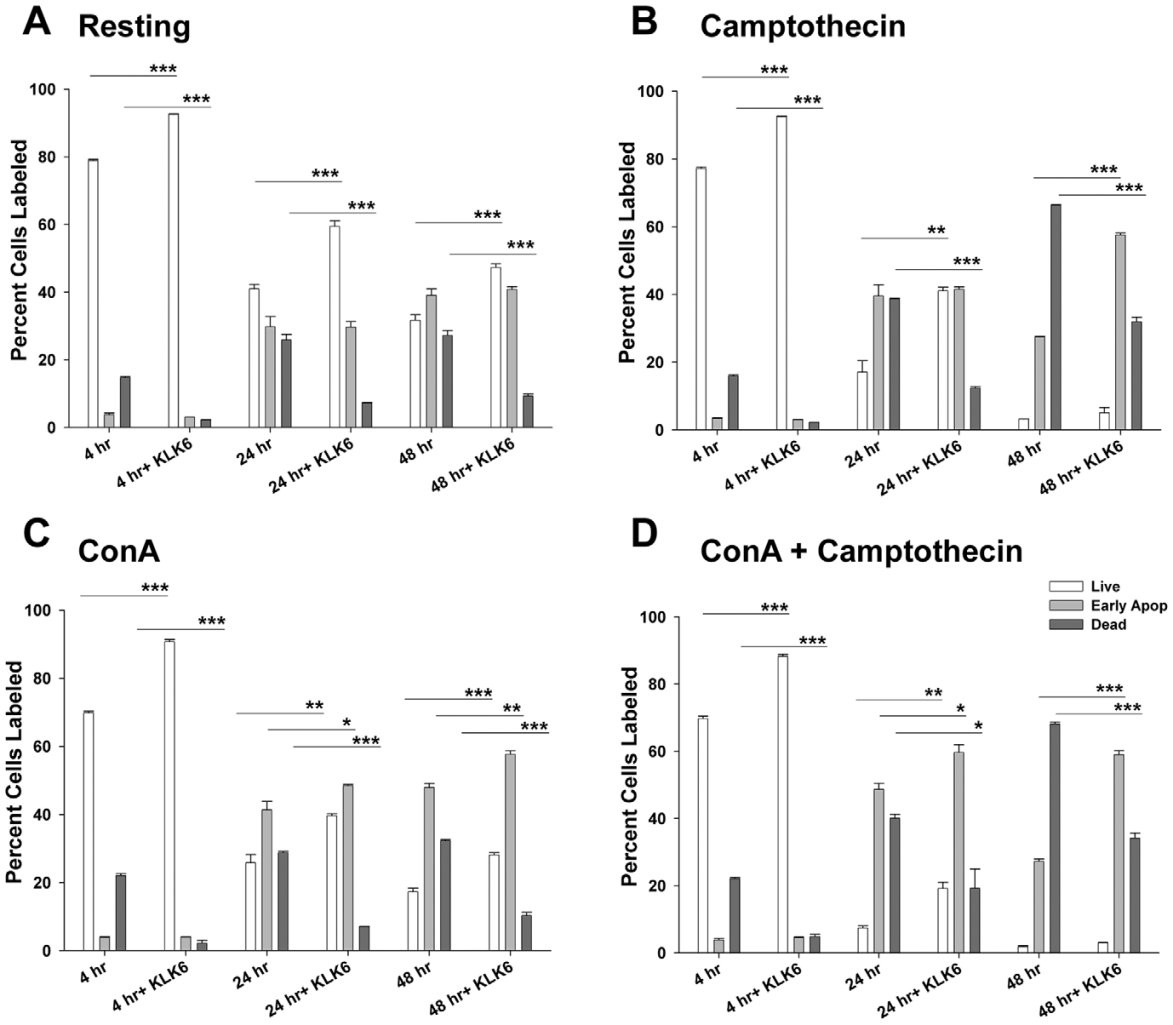
KLK6 promotes survival of Jurkat T cells in part by slowing the apoptotic cascade. To address the effect of KLK6 on apoptosis, Jurkat T cells were grown under resting conditions (A), or in the presence of apoptosis inducing agents (B to D), then labeled with Annexin V-PE and 7AAD prior to flow cytometry. Under resting conditions (A), and in the presence of camptothecin (B, 1.0 µM), ConA (C, 5 µg/ml), or ConA plus camptothecin (D), KLK6 (10 µg/ml) promoted an increase in the percentage of live cells (unlabeled) and a decrease in the percentage of dead cells (Annexin V^+^ and 7AAD^+^) at both acute (4 hr), subacute (24 hr), and in some cases the more chronic time point examined (48 hr). Interestingly, in the presence of apoptosis inducing agents (B to D), KLK6 also promoted a corresponding increase in the percentage of Jurkat T cells positive for Annexin V, but negative for 7AAD, and therefore classified as in the early stages of apoptosis. These results suggest KLK6 may delay but not block the apoptotic cascade. Data shown are for triplicate cultures examined in parallel and are expressed as mean ± SEM, One Way ANOVA with SNK post hoc test for multiple comparisons; P<0.001***, P≤0.005**, P≤0.02*. All results shown are representative of those seen across at least three experiments using independent cell culture preparations.

**Figure 5:**
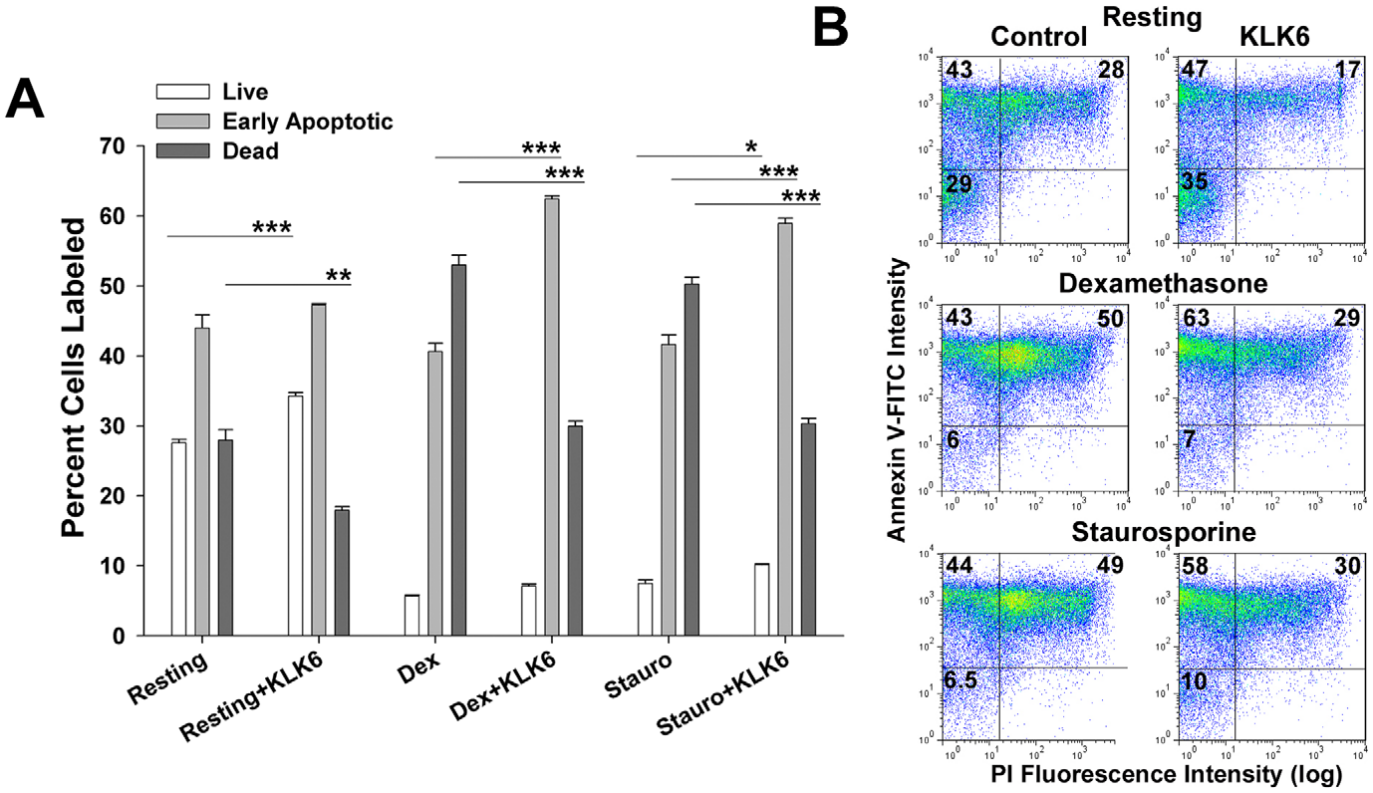
The ability of KLK6 to decrease death of murine splenocytes relates to its ability to delay the apoptotic cascade. (A) Histogram and corresponding dot plots (B) demonstrate that KLK6 significantly reduced the percentage of dead cells (Annexin V-FITC^+^ and PI^+^) observed in cultures of splenocytes under resting conditions and after exposure to dexamethasone (0.1 µM), or staurosporine (1 µM). Reduced cell death was accompanied by a significant increase in the live population (unlabeled cells) under resting conditions and with exposure to staurosporine. KLK6 exposure also resulted in a corresponding increase in the percentage of early apoptotic cells (Annexin V-FITC^+^, PI^-^) in the presence of apoptosis inducing agents. Data shown are the mean ± SEM of triplicate cultures examined in parallel; One Way ANOVA with SNK post hoc test for multiple comparisons, P<0.001***, P<0.003**, P<0.007*. All results shown are representative of that seen in at least 3 independent experiments.

**Figure 6:**
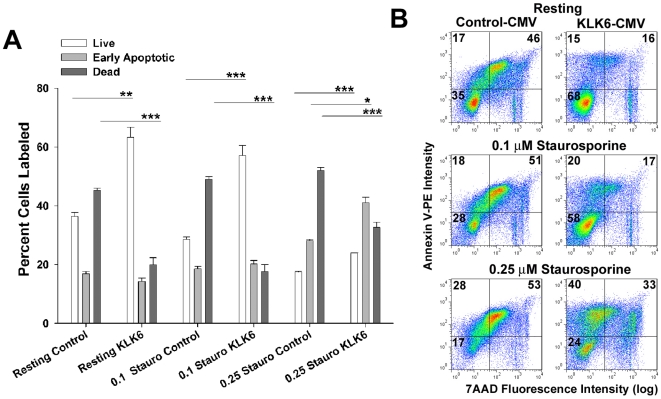
KLK6 over expression in Jurkat T cells reduces cell death. Jurkat T cells were stably transduced with a vector in which the human KLK6 gene is constitutively expressed under the control of a CMV promoter, or with an empty vector (Control), and levels of live (Annexin V-PE^-^ and 7AAD^−^), early apoptotic (Annexin V-PE^+^ and 7AAD^−^), or dead (Annexin V-PE^+^ and 7AAD^+^) cells determined by flow cytometry under resting conditions, or after 24 hr periods of exposure to 0.1 or 0.25 µM staurosporine. (A) Histogram and corresponding dot plots (B), demonstrate KLK6 over expression produces effects largely parallel to those afforded by treatment of cultures with recombinant KLK6. There was a decrease in the percentage of dead cells and an increase in the number of live cells in Jurkat T cells over expressing KLK6, relative to those expressing an empty vector. KLK6 over expression also reduced cell death in the presence of 0.1 or 0.25 µM stuarosporine relative to that seen in cells stably transduced with empty vector. Reductions in cell death were likely to reflect in part a delay in apoptosis, since after 24 hr exposure to 0.25 µM Staurosporine, KLK6 over expression not only reduced the number of dead cells and increased the number of live cells, but cells in the early stages of apoptosis (AnnV-PE^+^, 7AAD^−^) were also significantly elevated. Data are expressed as mean ± SEM of triplicate cultures examined in parallel; One Way ANOVA with SNK post hoc test for multiple comparisons, P<0.001***, P = 0.002**, P = 0.003*. All data shown is representative of that seen in at least 3 independent cell culture experiments.

**Figure 7:**
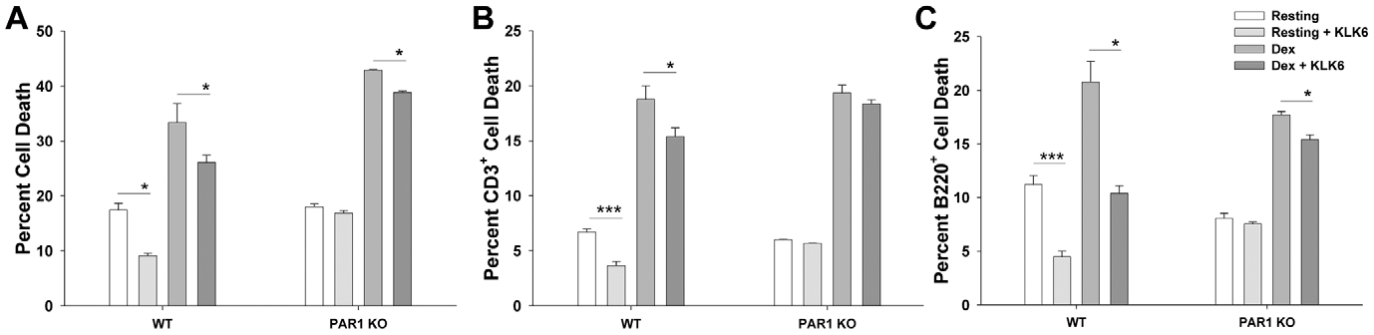
The ability of KLK6 to reduce murine T and B cell death depends in part on activation of PAR1. To determine the involvement of PAR1 in KLK6-mediated reductions in lymphocyte death, the ability of KLK6 (10 µg/ml) to reduce cell death under resting conditions, or in response to dexamethasone (0.1 uM), was compared between wild type and PAR1^−/−^ splenocytes. Comparisons were made between the total percentage of 7AAD^+^ dead cells, or between those additionally labeled to recognize CD3^+^ T cells, or B220^+^ B cells. (A) KLK6 (10 µg/ml) significantly reduced the overall percentage of 7AAD^+^ dead cells under resting conditions and in response to dexamethasone (0.1 µM). KLK6-mediated reductions in overall cell death in both paradigms (A) were seen to encompass both CD3^+^ T lymphocytes (B) and B220^+^ B lymphocytes (C). The absence of PAR1 (PAR1 KO) impaired the ability of KLK6 to significantly reduce the overall percentage of cell death and that associated with T cells and B cells under resting conditions. The absence of PAR1 also impaired the ability of KLK6 to reduce dexamethasone-induced CD3^+^ T cell death. Data shown are mean ± SEM of triplicate cultures examined in parallel; One Way ANOVA with SNK post hoc test for multiple comparisons, P = 0.002***, P≤0.003**, P<0.05*. Results shown are representative of that seen in at least 3 independent experiments.

**Figure 8:**
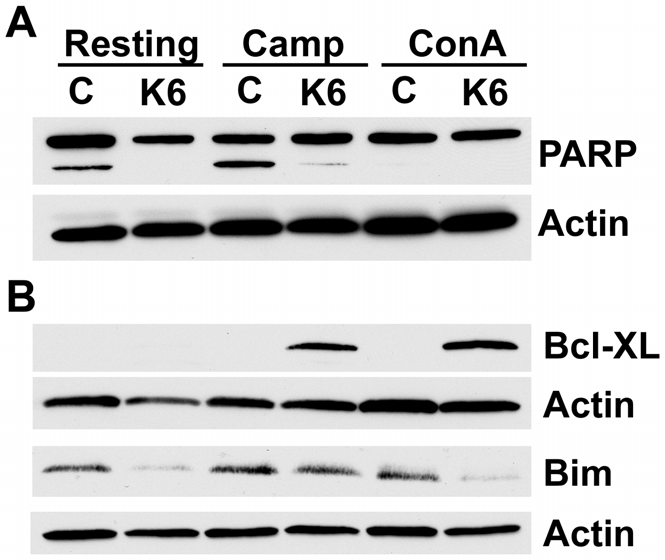
KLK6 differentially regulates PARP cleavage and Bcl-2 family member protein levels. Western blots illustrate KLK6 (10 µg/ml)-induced changes in the amount of cleaved PARP (116 kDa intact form, 89-kDa fragment, A), or in levels of Bcl-2 family members (B), observed in murine splenocytes derived from WT mice and cultured for a 24 hr period under resting conditions, or in the presence of camptothecin (1.0 µM), or ConA (5 µg/ml). Relative to non-KLK6-treated control samples, KLK6-treatment promoted reductions in PARP cleavage in WT resting (98% reduction) and camptothecin (89% reduction) co-treated cultures (A). Significant PARP cleavage was not seen with ConA treatment at the 24 hr time point examined. B, The pro-survival protein Bcl-XL was elevated in cultures exposed to camptothecin or ConA when co-treated with KLK6. Relative to non-KLK6 treated sister wells, detection of Bcl-XL was elevated by 40-fold in the presence of KLK6+camptothecin and 80-fold in the presence of ConA+KLK6. KLK6-induced changes in Bcl-XL were not seen in resting cultures. KLK6 suppressed detection of the pro-apoptotic protein Bim by 83 and 84% in resting and Con A treated cultures respectively, and by 16% in cultures treated with camptothecin. Actin was used to control for loading in each case. All Western blots shown are representative of from 2–5 separate cell culture experiments.

As a first approach to define the possible immunological actions of KLK6, its effects on survival and proliferation of murine splenocytes were examined and compared to another kallikrein family member, KLK1. While KLK6 did not significantly alter splenocyte proliferation after 24 or 72 hr, it did promote a substantial and dose dependent reduction in the number of cells positive for markers of cell death across the same time points (Fig. 1, see also Figs. 2, 3, 4, 5, 6, 7, 8). Significant reductions in the number of PI positive dead cells were observed under resting conditions when splenocytes were cultured in the presence of either 1 or 10 µg/ml of KLK6 at the time of plating. Since 10 µg/ml of KLK6 generated the most robust rescue this concentration was carried forward in all subsequent experiments. Also, KLK6 promoted cell survival at both 24 and 72 hr time points such that most subsequent experiments were carried out to 24 hr. Parallel examination of a second kallikrein, KLK1, generated by baculoviurs expression in parallel with KLK6 [26], did not significantly impact cell survival. KLK1 (10 µg/ml) did however promote a significant increase in the percent of divided cells when examined 24 hr post-stimulation. Significant differences in proliferation were not seen at lower concentrations of KLK1, or when cells were examined at 72 hr. The significance of KLK1's mitogenic effects toward splenocytes will need to be followed up in future studies. The general lack of KLK6-induced proliferative effects support the hypothesis that overall changes in splenocyte survival and death observed across experiments were not due to KLK6-mediated changes in cell proliferation.

### KLK6 promotes survival of T cells and B cells across multiple cell death paradigms

To determine whether promotion of lymphocyte survival represents a fundamental feature of KLK6 biology, we examined its ability to reduce cell death across a range of paradigms. KLK6 not only reduced the death of splenocytes which occurs after explant and culture (Resting, Figs. 1, 2, 3, 4, 5, 6, 7), but also that seen in response to 0.1 µM dexamethasone (Figs. 2, 3, 7). KLK6 also reduced the death of splenocytes induced by 1 µM staurosporine (Fig. 5) and in response to 2 µg/ml Fas ligand (Fig. 2). Co-labeling of splenocytes with CD3 to label T lymphocytes, and B220 to label B lymphocytes, in conjunction with 7AAD to identify cell death, indicated that KLK6 reduced the number of dead cells in both populations in resting cultures and in response to dexamethasone (Fig. 7). Parallel to observations using mixed splenocyte preparations, and supporting strong KLK6-mediated effects toward T lymphocytes, recombinant KLK6 also significantly diminished Jurkat T cell death under resting conditions and when exposed to 1 µM camptothecin or 1 µM camptothecin plus 5 µg/ml ConA, and in response to Fas ligand receptor cross linking (Figs. 2, 4). The ability of KLK6 to rescue splenocytes or Jurkat T cells across cell death paradigms was consistently observed when KLK6 was applied simultaneously with death inducing agents. A two hour pre-incubation with KLK6 prior to dexamethasone application was shown to result in a small but statistically significant enhancement of survival (Fig. 2, P<0.05).

To determine whether prolonged or continual exposure to KLK6 was necessary to promote significant rescue effects, we compared the pro-survival effects of abbreviated periods of KLK6 stimulation (Fig. 3). Interestingly, the pro-survival effects of KLK6 over a 24 hr period under resting conditions or in the presence of dexamethasone were similar when whole splenocyte cultures were treated with KLK6 for 5, 30 or 60 min, or for the full 24 hr period of culture. However when experimental endpoints were extended to 48 hr, only continual treatment with KLK6, but not transient exposure up to 60 min, was sufficient to promote more prolonged pro-survival effects.

In addition to examination of the effects of recombinant KLK6, we additionally determined whether over expression of the KLK6 gene in the Jurkat T cell line would similarly alter the extent of cell death observed (Fig. 6). Jurkat cells were stably transduced with a vector in which the KLK6 gene is constitutively expressed under control of the CMV promoter, or empty vector, and KLK6 expression levels quantified by real time PCR [3]. Similar to KLK6 transcript levels we have previously reported in Jurkat T cells, mean KLK6 transcript levels (± SEM) were 5.5E+05 ±2.5E+04 in cells transduced with vector alone and 2.65E+07 ± 1.0E+04 in KLK6-CMV transduced cells. GAPDH transcript levels in the same samples were 4.0E+06 ± 1.7E+05 and 3.9E+06 ± 1.8E+05, respectively, demonstrating equal RNA loading. This represents a 50-fold increase in KLK6 expression levels, a result replicated by PCR more than 3 times using separate cell culture preparations. Importantly, relative to cells transduced with vector alone, those transduced with the KLK6-CMV vector showed a significant increase in the number of live cells (7AAD^−^, Annexin V^-^), and a decrease in the number of dead cells (7AAD^+^) under resting conditions, and when challenged with 0.1 or 0.25 µM staurosporine (Fig. 6).

### KLK6 affects the apoptotic cascade

The ability of KLK6 to specifically affect apoptosis in splenocytes and Jurkat T cells was assessed by comparing KLK6-induced changes in the relative percentage of live cells (AnnexinV^-^, PI^−^), early apoptotic cells (AnnexinV^+^, PI^−^), and dead cells (AnnexinV^+^, PI^+^) across several cell death paradigms (Figs. 4, 5, 6). In some cases 7AAD was used in place of PI.

In the case of Jurkat T cells under resting conditions, recombinant KLK6 promoted a reduction in the number of dead cells and a corresponding increase in the number of live cells, but did not significantly alter the number of early apoptotic cells when examined at 4, 24 or 48 hr post plating (Fig. 4). When Jurkat cells were challenged with the topoisomerase inhibitor camptothecin, with ConA, or a combination of these agents, KLK6 reduced the number of dead cells at the 4, 24 and 48 hr time points examined, mirroring observations under resting conditions. A significant increase in the number of live cells was also seen in each circumstance except after 48 hr exposure to the Camptothecin or the ConA+Camptothecin combination. Interestingly, Jurkat cells cultured in the presence of KLK6 in addition to ConA, or ConA plus camptothecin, showed an accumulation of cells at early apoptotic stages at the 24 hr time point examined. By 48 hr, KLK6-induced accumulation of early apoptotic cells was seen under all three death-inducing conditions, including camptothecin alone. Supporting the concept that KLK6 is able to delay apoptosis under certain conditions, KLK6-CMV over expression in Jurkat cells was also associated with not only an increase in the number of live cells and a decrease in the overall number of dead cells, but in the presence of 0.25 µM staurosprorine, a significant accumulation of AnnexinV^+^, 7AAD^−^) early apoptotic cells was also observed (Fig. 6).

Examination of live, early apoptotic, and dead cells in murine splenocyte cultures generated results parallel to those observed in Jurkat T cells, that is KLK6-induced decreases in the percentage of dead cells was accompanied by increases in the number of cells in the live and/or early apoptotic populations depending on the conditions examined (Fig. 5). Under resting conditions over 24 hr, the presence of KLK6 significantly reduced the number of dead cells with a corresponding increase in the live population, while the number of early apoptotic cells was largely unchanged. In the presence of dexamethasone (0.1 µM), or staurosporine (1 µM), co-exposure to KLK6 promoted a significant decrease in the number of dead cells and a small but significant increase in the live cell population. In addition, under these death-inducing conditions, exposure to KLK6 also resulted in a significant accumulation of cells at the early apoptotic stage.

### KLK6-mediated lymphocyte survival occurs in T cells and B cells and depends in part on activation of PAR1

To investigate the pro-survival effects mediated by KLK6 toward splenic lymphocytes, and the potential involvement of PAR1, we examined the ability of KLK6 to alter resting or dexamethasone-induced death in whole splenocyte cultures isolated from WT or PAR1 knockout mice and used flow cytometry approaches to specifically examine the response of T and B cell subsets (Fig. 7). The overall extent of cell death (see also Figs. 1, 2, 3, 5), in addition to that occurring specifically in splenic CD3^+^ T cells, and B220^+^ B cells, was examined. Basal levels of cell death were not observed to differ significantly between WT and PAR1^−/−^ mice. Under resting conditions, the absence of PAR1 completely blocked the ability of KLK6 to support the survival of splenocytes overall, and in either of the CD3^+^ T cell or B220^+^ B cell subsets. In the case of dexamethasone treatment of WT splenocytes, KLK6 was seen to significantly reduce overall cell death and that observed in both CD3^+^ T cells and B220^+^ B cells. In splenocytes isolated from PAR1^−/−^ mice, KLK6 was no longer able to significantly decrease dexamethasone induced CD3^+^ T cell death, although overall cell death and that of B220^+^ B cells continued to be significantly reduced by KLK6.

The ability of KLK6 to alter apoptosis was further addressed by examining its effects on cleavage of PARP, one of the final caspase substrates cleaved in the apoptotic cascade, in whole splenocyte preparations under resting conditions or when treated with camptothecin or ConA (Fig. 8A). Significant levels of cleaved PARP were detected in WT splenocytes grown under resting conditions and when exposed to camptothecin for 24 hr. In each case, co-treatment with KLK6 significantly reduced the amount of cleaved PARP detected by Western blot.

### KLK6 differentially regulates Bcl-2 family member signaling

To begin to address the mechanism by which KLK6 may promote splenocyte survival we examined its effects on Bcl-2 family pro- and anti-apoptotic signaling cascades. Levels of the pro-survival protein Bcl-XL in resting, camptothecin or ConA treated whole splenocyte cultures were below the detection limit of our assay. When splenocytes were exposed to either camptothecin or ConA in the presence of KLK6 however, significant elevations in Bcl-XL were observed (Fig. 8B). KLK6 did not elevate Bcl-XL protein levels in resting splenocyte cultures. Interestingly, KLK6 reduced levels of the pro-apoptotic protein BIM, under resting conditions and in the presence of camptothecin or ConA (Fig. 8B).

## Discussion

KLK6 levels are elevated at sites of inflammation in active MS lesions, in CNS trauma, and in animal models of these pathologies [1], [46]. Elevations are due in part to expression of KLK6 by infiltrating immune cells [1], [5], [46], yet very little is currently known regarding the role of this newly identified serine protease in immune function. In this study, we demonstrate that KLK6 exerts pro-survival effects toward murine splenocytes and Jurkat T cells across a range of cell death paradigms. We further demonstrate that the pro-survival effects of KLK6 in splenocytes depend in part on activation of the G-protein coupled receptor, PAR1, and involve at least a subset of Bcl-2 family member signaling cascades. Taken together, these findings delineate KLK6 as a new player within the network of regulatory mechanisms that govern survival of immune cells.

The present studies demonstrate for the first time that KLK6 is able to promote survival of murine splenocytes as well as the human leukemic Jurkat T cell line across divergent cell death paradigms encompassing both the intrinsic/mitochondrial and the extrinsic Fas-Fas ligand-induced apoptotic signaling pathways. The ability of KLK6 to promote survival of T-lymphocytes is supported by observations of its pro-survival effects toward both Jurkat T cells and CD3 positive cells in whole splenocyte cultures. Notably, we were able to demonstrate robust pro-survival effects of KLK6 toward Jurkat T cells when cells were either treated with recombinant KLK6 or when KLK6 over expression was enforced using a KLK6 expression construct under control of a CMV promoter. The likely fundamental role of KLK6 in lymphocyte survival in general is supported by findings in whole splenocyte cultures that pro-survival effects extend also to B220 positive B-lymphocytes. Contrasting its robust pro-survival effects, KLK6 did not significantly alter splenocyte proliferation under the conditions of this study, indicating that proliferative effects were unlikely to account for its ability to increase the number of viable cells. Interestingly, another member of the KLK family, KLK1, better known as true tissue kallikrein, did exert proliferative effects toward splenocytes in the first 24 hr period of culture. Taken with evidence that both KLK1 and KLK6 are elevated in the serum of MS patients [4], it is tempting to speculate that these proteases may operate in tandem to support inflammation by uniquely enhancing immune cell survival and proliferation.

Experiments examining Annexin V labeling, which is taken as a marker of the early stages of apoptosis, in conjunction with markers of plasma membrane permeabilization, such as PI or 7AAD that are indicative of cell death, suggest KLK6 is capable of delaying the cell death cascade at early stages of apoptosis. While KLK6 increased the number of live cells and decreased the detection of dead cells across a range of cell death paradigms, both in whole splenocyte preparations and in the case of Jurkat T cells, KLK6 also promoted a significant accumulation of cells at the early stages of apoptosis (Annexin V^+^, PI^−^). The accumulation of Annexin V positive cells accompanied by a decrease in dead cells was particularly prominent in Camptothecin and ConA treated Jurkat cells carried out to the 48 hr time point. These data favor a model whereby KLK6 delays the apoptotic process. Taken with findings herein that KLK6 also blocks the cleavage of PARP, it is likely that KLK6 delays the apoptotic cascade from entering into the effector phase when caspase 3 activation and PARP cleavage occur.

The mechanism by which KLK6 promotes lymphocyte survival was shown to involve, at least in part, activation of the thrombin receptor PAR1. Using genetically PAR1 deficient mice the present studies demonstrate that the absence of PAR1 completely blocks the ability of KLK6 to reduce the final stages of death of T- and B-lymphocytes under resting conditions. While there is growing evidence regarding the role of PAR activation in inflammation [47], [48], particularly with regard to roles in driving cytokine production and proliferation, this is the first report of the likely involvement of PAR1 activation in the control of lymphocyte survival. Of interest in this regard, several recent studies have demonstrated a role for PAR1 in survival of non-immune cells including, prostate cancer cells via NF_K_B and activation of Bcl-XL [49], breast cancer cells via activation of AKT [50], CC139 fibroblasts by suppression of Bim [51], and rat astrocytes by release of chemokine growth-regulated oncogene/cytokine-induced neutrophil chemoattractant-1 [52]. The present *in vitro* results suggest that the anti-inflammatory effects of PAR1 deficiency, already described in some murine models, may relate in part to the absence of pro-survival signaling mediated by certain agonists such as KLK6 at this receptor, and therefore increased vulnerability of lymphocytes to apoptosis. For example, PAR1 deficiency has been associated with attenuated inflammatory responses in murine crescentic glomerulonephritis [20], colitis [19], and BSA-induced arthritis [21]. Treatment of mice with the thrombin inhibitor hirudin, also blocks inflammation in crescentic glomerulonephritis and in addition was recently shown to attenuate disease severity in EAE [53].

The absence of PAR1 had less effect on the ability of KLK6 to promote overall splenocyte survival, or that of B lymphocytes in the presence of dexamethasone compared to cells under resting conditions, although its absence did significantly reduce KLK6-mediated survival of CD3^+^ T lymphocytes. These differential effects depending on the cell death paradigm examined suggest the involvement of additional KLK6-mediated pro-survival mechanisms when cells are under stress and that these may additionally be cell type specific. Given our prior studies that show KLK6 is also capable of activating PAR2 [8], [9], [10], [11], as well as bradykinin receptor 2 [11], it is highly possible that KLK6-mediated pro-survival effects occur by activation of multiple receptors thereby triggering signaling cascades that combine to overcome or delay death signaling cascades. In this way, the ability of KLK6 to regulate survival of T and B cells would be sharply tuned by differential expression of receptors activated by KLK6 in response to changing environmental cues.

That KLK6-mediates its pro-survival effects in a receptor dependent fashion is also supported by observations herein which show as little as 5 min exposure to KLK6 was sufficient to drive survival in the short term (i.e. 24 hr). Of interest in this regard, only continual KLK6 exposure, but not short pulses up to 60 min in duration, were able to delay cell death over more prolonged culture periods.

KLK6 was shown to affect the balance of pro- and anti-apoptotic members of the Bcl-2 family. In whole splenocyte populations challenged with camptothecin or ConA, KLK6 up regulated the pro-survival protein Bcl-XL and suppressed levels of the pro-apoptotic BH3 only family member Bim. Interestingly, while KLK6 suppressed Bim in whole splenocyte cultures under all conditions examined, its ability to up regulate Bcl-XL was only detected in cells undergoing an additional stress, such as camptothecin or ConA, but not in resting cells. These results support the concept that KLK6 promotes survival in part by the integration of multiple signaling pathways, including those seen only in the face of cellular stress. While much remains to be learned regarding the signaling pathways triggered by KLK6 to alter immune cell survival under different conditions, the ability of KLK6 to strongly suppress Bim is of particular interest since this BH3 only family member is known to play an essential role in lymphoid and myeloid cell homeostasis [54], in the deletion of autoreactive T [55] and B cells [56], in addition to the shut down of acute T cell immune responses [33], [57]. Given the elevated levels of KLK6 in MS patient serum and in active MS lesions we hypothesize one role may be to prolong inflammatory cell survival by altering levels of Bcl-2-family members. The current findings point to the need for additional studies to dissect the signaling cascades elicited by KLK6 in different immune cell subsets and its impact on their functional properties *in vivo*.

Collectively, these *in vitro* studies define KLK6 a possible new participant in the molecular signaling cascades that regulate immune cell life and death. Given the relatively widespread distribution of KLK6 in human tissues [3], [58], [59], and increasing awareness of its regulation in disease [1], [4], [28], [58], [60], [61], [62], [63], this novel enzyme is in a position to play an important regulatory role in immune-related disorders, including but not limited to MS. Since insufficient lymphocyte apoptosis is a well known participant in autoimmune and lymphoproliferative disease, but in excess can promote immunodeficiency, approaches that target KLK6 may hold new promise for the treatment of diseases with an inflammatory substrate.
